# Supplementary material for: Imaging-Guided Evaluation of the Novel Small-Molecule Benzosuberene Tubulin-Binding Agent KGP265 as a Potential Therapeutic Agent for Cancer Treatment
Source: Cancers (Basel). 2021 Sep 24;13(19):4769. doi: 10.3390/cancers13194769 (PMC8507561; doi:10.3390/cancers13194769)
Supplement: Supplementary file 1 [file cancers-13-04769-s001.zip › cancers-1340749-supplementary.pdf]

Supplementary Materials

Imaging-Guided Evaluation of the Novel Small-Molecule Benzosuberene Tubulin-Binding Agent KGP265 as a Potential Therapeutic Agent for Cancer Treatment

Yihang Guo, Honghong Wang, Jeni L. Gerberich, Samuel O. Odutola, Amanda K. Charlton-Sevcik, Maoping Li, Rajendra P. Tanpure, Justin K. Tidmore, Mary Lynn Trawick, Kevin G. Pinney, Ralph P. Mason and Li Liu

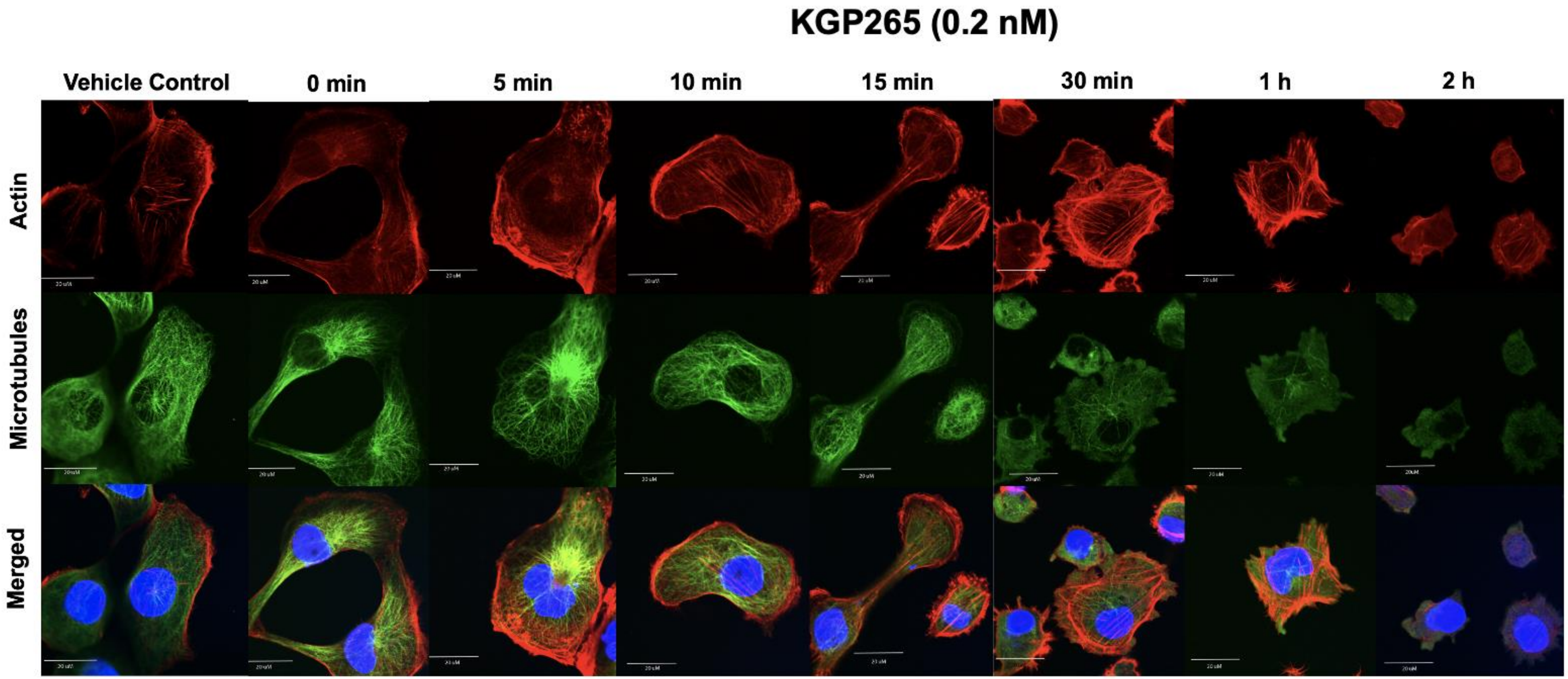

Figure S1. Time course of MDA-MB-231 cells treated with KGP265. Microtubule disruption (15–30 min) was followed by actin stress fiber formation (30 min–2 h). An increasing number of cells demonstrated rounding, contraction, blebbing, and detachment over the course of 2 h with the treatment of MDA-MB-231 cells with 0.2 nM KGP265. Confocal microscopy of representative cells stained for actin, microtubules, and nuclei. All imaging were captured under confocal oil immersion lens 60×, scale bar: 20μm.

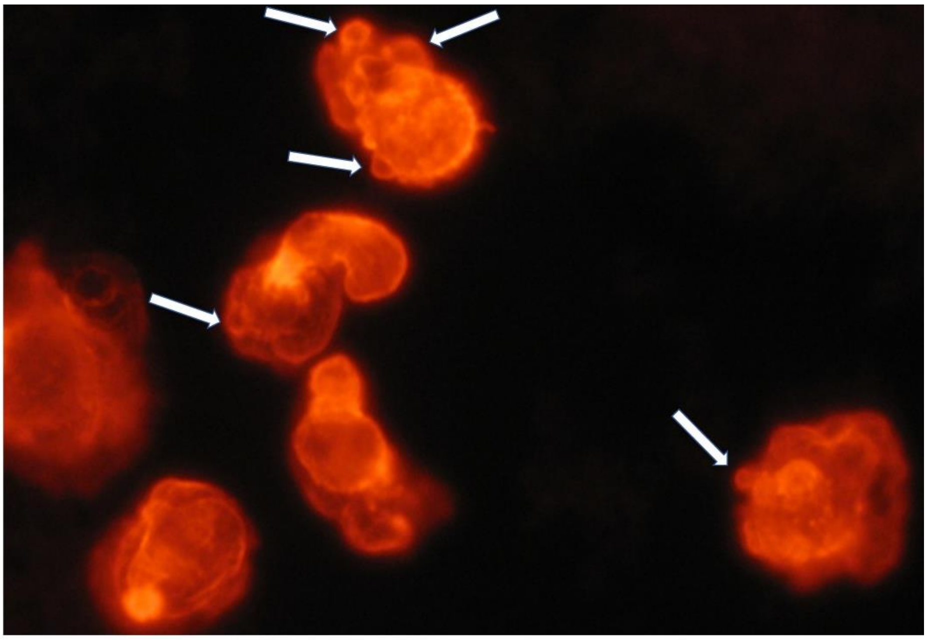

Figure S2. Confocal microscopy of MDA-MB-231 cells treated with KGP18. Extensive blebbing (white arrows), rounding, contraction and detachment were observed in MDA-MB-231 cells treated with 2.8 nM KGP18 for 2 h and stained for actin, This imaging was captured under confocal oil immersion lens 60×.

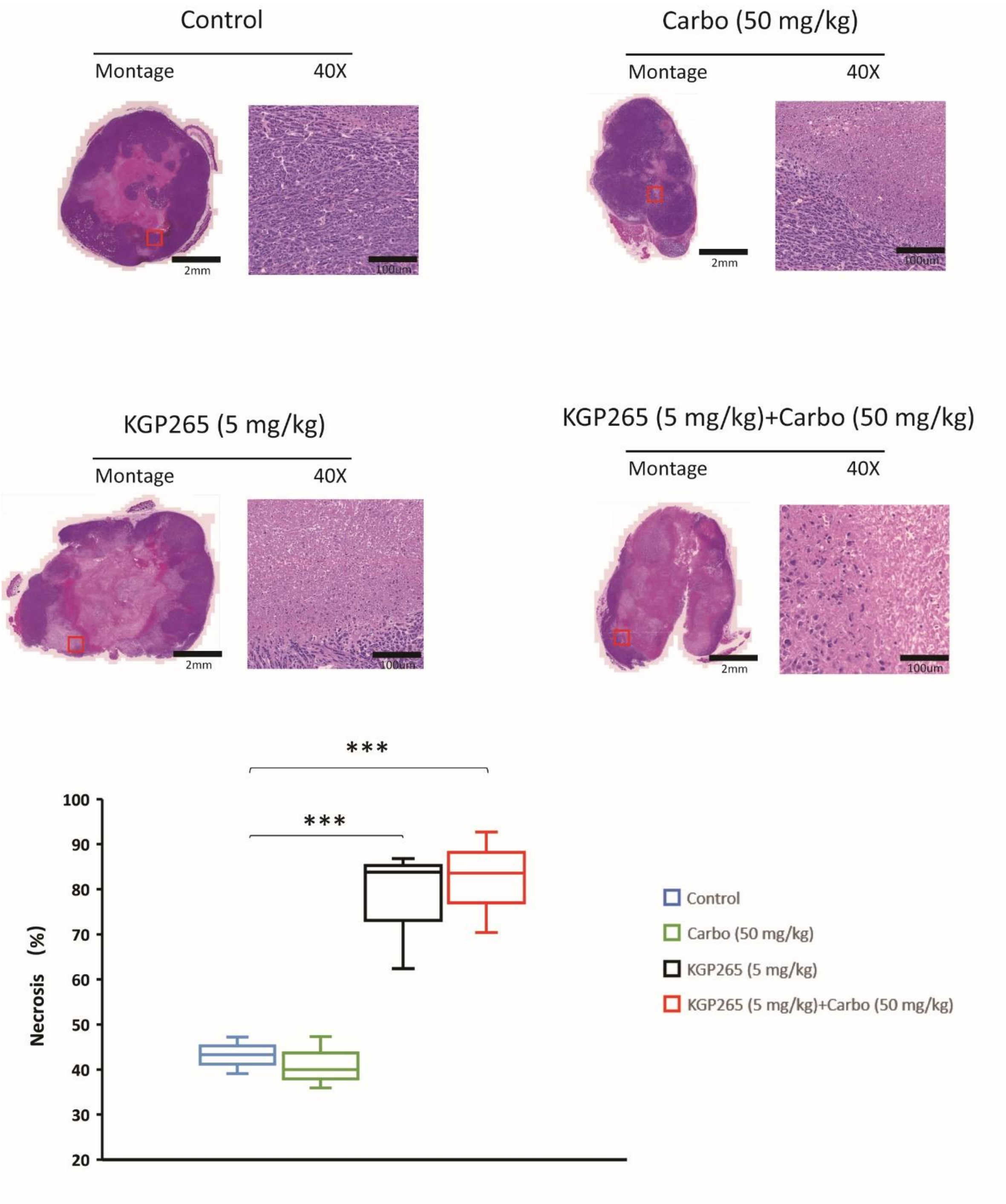

Figure S3. Histological analysis of MDA-MB-231 tumors in response to Control saline, Carboplatin (Carbo), KGP265 and KGP265 with Carboplatin. The red rectangles in the montages show expanded regions in 40× images. For the statistical analysis, the levels in the treated tumors were compared to the levels in the control tumors with a two-way unmatched ANOVA test. ***, $p < 0.001$.

© 2021 by the authors. Licensee MDPI, Basel, Switzerland. This article is an open access article distributed under the terms and conditions of the Creative Commons Attribution (CC BY) license (http://creativecommons.org/licenses/by/4.0/).
